# A case study of a liver transplant-treated patient with glycogen storage disease type Ia presenting with multiple inflammatory hepatic adenomas: an analysis of clinicopathologic and genetic data

**DOI:** 10.1186/s12920-024-01888-6

**Published:** 2024-05-06

**Authors:** Ao Wang, Jiamei Wu, Xiaohui Yuan, Jianping Liu, Changli Lu

**Affiliations:** 1 Department of Pathology, West China Hospital, Sichuan University, Chengdu, 610000 China; 2 Department of Pathology, Affiliated Hospital of Panzhihua University, Panzhihua, 617000 China

**Keywords:** GSD-Ia, Hepatic adenomas, G6PC gene, Liver transplant-treated, Clinicopathologic

## Abstract

**Background:**

Glycogen storage disease (GSD) is a disease caused by excessive deposition of glycogen in tissues due to genetic disorders in glycogen metabolism. Glycogen storage disease type I (GSD-I) is also known as VonGeirk disease and glucose-6-phosphatase deficiency. This disease is inherited in an autosomal recessive manner, and both sexes can be affected. The main symptoms include hypoglycaemia, hepatomegaly, acidosis, hyperlipidaemia, hyperuricaemia, hyperlactataemia, coagulopathy and developmental delay.

**Case presentation:**

Here, we present the case of a 13-year-old female patient with GSD Ia complicated with multiple inflammatory hepatic adenomas. She presented to the hospital with hepatomegaly, hypoglycaemia, and epistaxis. By clinical manifestations and imaging and laboratory examinations, we suspected that the patient suffered from GSD I. Finally, the diagnosis was confirmed by liver pathology and whole-exome sequencing (WES).

WES revealed a synonymous mutation, c.648 G > T (p.L216 = , NM_000151.4), in exon 5 and a frameshift mutation, c.262delG (p.Val88Phefs*14, NM_000151.4), in exon 2 of the G6PC gene. According to the pedigree analysis results of first-generation sequencing, heterozygous mutations of c.648 G > T and c.262delG were obtained from the patient's father and mother.

Liver pathology revealed that the solid nodules were hepatocellular hyperplastic lesions, and immunohistochemical (IHC) results revealed positive expression of CD34 (incomplete vascularization), liver fatty acid binding protein (L-FABP) and C-reactive protein (CRP) in nodule hepatocytes and negative expression of β-catenin and glutamine synthetase (GS). These findings suggest multiple inflammatory hepatocellular adenomas. PAS-stained peripheral hepatocytes that were mostly digested by PAS-D were strongly positive.

This patient was finally diagnosed with GSD-Ia complicated with multiple inflammatory hepatic adenomas, briefly treated with nutritional therapy after diagnosis and then underwent living-donor liver allotransplantation.

After 14 months of follow-up, the patient recovered well, liver function and blood glucose levels remained normal, and no complications occurred.

**Conclusion:**

The patient was diagnosed with GSD-Ia combined with multiple inflammatory hepatic adenomas and received liver transplant treatment. For childhood patients who present with hepatomegaly, growth retardation, and laboratory test abnormalities, including hypoglycaemia, hyperuricaemia, and hyperlipidaemia, a diagnosis of GSD should be considered. Gene sequencing and liver pathology play important roles in the diagnosis and typing of GSD.

## Background

Glycogen storage diseases (GSDs) are a group of congenital genetic metabolic diseases caused by defects in various enzymes involved in glycogen metabolism. It is characterized by excessive deposition of glycogen in various organs and tissues of the body, mainly involving the liver and/or muscle, and is a relatively rare metabolic disease. According to different enzyme defects or transporters, GSD can be divided into more than 10 types, and type Ia is relatively common. GSD-Ia (OMIM #232,200), which accounts for approximately 80% of GSD-I, is an autosomal recessive metabolic disorder caused by a deficiency in glucose-6-phosphatase-α (G6 Pase-α or G6PC) [1–4]. G6 Pase-α catalyses the hydrolysis of glucose-6-phosphate (G6P) to glucose and phosphate in the terminal step of gluconeogenesis and glycogenolysis and is a key enzyme for endogenous glucose production [5–7]. The incidence of GSD-Ia is approximately 1 in 100,000 to 400,000 births, and it accounts for approximately 25% of all types of glycogen storage disease [8]. Patients affected by GSD-Ia are unable to maintain glucose homeostasis and present with fasting hypoglycaemia, hepatomegaly, nephromegaly, hyperlipidaemia, hyperuricaemia, lactic acidaemia, and growth retardation [9]. The diagnosis of this disease mainly depends on clinical manifestations and laboratory examination, assisted by computerized tomography (CT), B-ultrasonography and liver pathology [10, 11]. Treatment includes symptomatic supportive therapy, enzyme replacement therapy, gene therapy and liver transplantation [12, 13]. In addition, hepatocellular adenoma and renal dysfunction are frequent late complications [14, 15]. Delayed diagnosis and inappropriate therapy can lead to many complications, such as growth failure, osteoporosis, refractory gout, renal failure, hepatocellular carcinoma (HCC), and pulmonary hypertension [16, 17]. Although the clinical examination has some specificity, the clinicopathologic and genetic analysis of this disease requires further study.

To improve the understanding of this disease, we report here a patient with GSD-Ia who was hospitalized at West China Hospital of Sichuan University in August 2022. This study was approved by the ethics committee of West China Hospital of Sichuan University, Chengdu, China. Informed consent was obtained from the patient’s parents. The study protocol conforms to the ethical guidelines of the 1975 Declaration of Helsinki.

## Case presentation

A 13-year-old female patient was admitted to the Liver Transplant Center, West China Hospital of Sichuan University, to identify the nature of the space-occupying lesions in her liver in August 2022. She had developed full-abdominal distention twelve years earlier without obvious cause. However, she had no fever, abdominal pain, diarrhoea, vomiting, melena, or yellowing of the skin, and she was not treated. Eight years ago, her parents found that the child’s growth and development were significantly behind those of her peers, with normal intelligence, abdominal distension, skin ecchymosis and epistaxis occurring approximately once a week. She was admitted to a local hospital and diagnosed with mucopolysaccharidosis syndrome but was not treated. One year prior, the child experienced syncope after physical exercise, which lasted for 2 min, and she woke up without headache, disturbance of consciousness or other discomfort. The patient presented to our hospital 5 months prior, and she was found to have hepatomegaly and multiple space-occupying lesions in the liver by B-ultrasonography and blood biochemical abnormalities. The doctor provided symptomatic treatment and ordered regular review. At this visit, her parents wanted to clarify the pathology of her liver for better treatment.

Physical examination revealed that this 13-year-old girl was 112 cm in height and 20.5 kg in weight, which are below the average height and weight of her peers. Her secondary sex characteristics were undeveloped, and she had facial signs of chronic liver disease. Her liver was palpable 6 cm below the right rib and at the level of the anterior superior iliac spine, with rigidity and a blunt edge. Moreover, the patient was a junior high school student with no menarche.

**Fig. 1:**
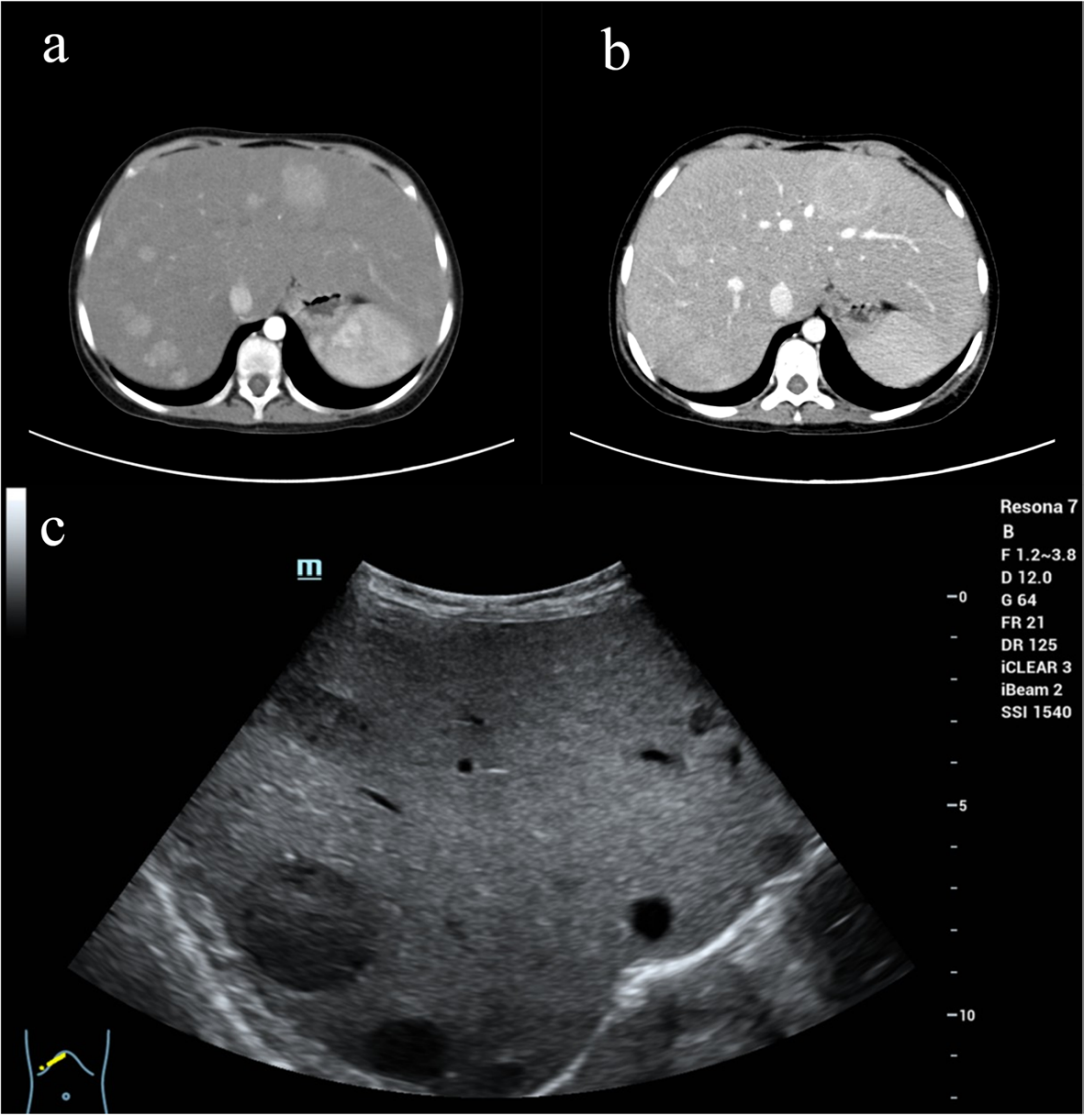
Imaging examination. **a** The arterial phase of the contrast-enhanced CT image shows multiple nodules and masses in the liver with significant enhancement. **b** The portal phase of the contrast-enhanced CT image shows that some nodules have mild and persistent enhancement. **c** B-ultrasonography showed multiple solid nodules in the context of fatty liver

Imaging studies showed evidence of multiple hepatic hyperplastic nodules with fatty liver changes. An upper abdominal enhancement CT scan showed that the liver volume was increased, the density of the liver parenchyma was decreased unevenly, there were multiple liver nodules and masses, the arterial phase was significantly enhanced, and the portal phase was mildly and continuously enhanced (Fig. 1a-b). B-ultrasonography revealed multiple solid nodules in the context of fatty liver. After contrast agent injection, the nodules showed high signal intensity in the arterial phase, a slightly greater signal intensity in the portal vein phase, and an enhanced parenchyma phase (Fig. 1c). Abdominal CT revealed a smooth surface of the ovary without abnormalities, but B-ultrasonography was not performed to further evaluate the development of the ovary.

Laboratory examinations revealed low glucose (1.83 mmol/L, reference range: 3.90–5.90 mmol/L), high lactic acid (11.71 mmol/L, reference range: < 2.10 mmol/L), high blood ammonia (48.4 μmol/L, reference range: 9.0–33.0 μmol/L), and high alanine transaminase (84 IU/L, reference range: < 40 IU/L) levels (Table 1). The levels of endocrine hormones were not measured for this patient.

**Table 1:** Examination data on presentation and follow-up

Variable	Reference range	First visit	8 Months (Before transplant)	12 Months (After transplant)
Height (cm)	–	111	112	113
Weight (kg)	–	19.5	20.5	20
BMI (kg/m^2^)	18.5–23.9	15.8	16.3	15.7
Glu (mmol/L)	3.90–5.90	2.39	1.83	4.11
LA (mmol/L)	< 2.10	7.77	11.71	3.88
BA (μmoi/L)	9.0–33.0	60.1	48.4	30.6
PYR (μmoi/L)	30–100	507.8	368.6	82.3
UA (μmoi/L)	160–380	595	574	121
TG (mmoi/L)	0.29–1.83	14.88	14.85	2.15
TCHO (mmoi/L)	2.80–5.70	8.79	8.21	5.11
β-HB (mmoi/L)	0.02–0.27	1.29	2.80	0.12
ALT (IU/L)	< 40	82	84	19
AST (IU/L)	< 35	179	160	39
GGT (IU/L)	< 45	254	333	367
Hepatic adenoma (min, max(cm))	–	0.6, 3.8	0.9, 4.1	–

*Glu* glucose, *LA* lactic acid, *BA* blood ammonia, *PYR* pyruvic, *UA* uric acid, *TG* triglyceride,*TCHO* total cholesterol, *β-HB* β hydroxybutyric acid, *ALT* alanine transaminase, *AST* aspartate transaminase, *GGT* γ-glutamyl transpeptidase, – no data

According to this evidence, she was suspected of suffering from glycogen storage disease (GSD). To confirm the suspicion of GSD, \ exome sequencing and liver biopsy were performed.

After informing the patient and family and obtaining signed informed consent, we collected the peripheral blood of the patient and her father and mother (her brother could not be collected for some of the reasons) and extracted DNA from white blood cells. We first sequenced the entire exome of the patient to identify the mutant gene and then used first-generation sequencing to verify the mutation in the patient and her family. Exome sequencing was performed via Illumina HiSeq using an Agilent Surelect Kit, and the platform for Sanger sequencing was an Applied Biosystems® 3730 DNA Analyser with a BigDyeTM Terminator v3.1 Cycle Sequencing Kit.

**Fig. 2:**
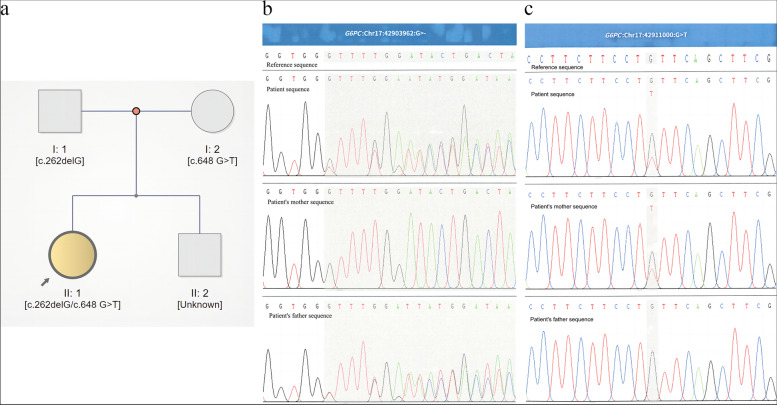
Mutational analysis of the patient pedigree. **a** The genotypes of the G6PC gene for family members. Roman numerals indicate generations, and Arabic numerals indicate individuals. Squares = males, circles = females. Affected individuals are denoted by yellow symbols, and unaffected individuals are denoted by grey symbols. The index patient is indicated by an arrow. The two mutations were inherited from the father (c.262delG) and mother (c.648 G > T), respectively. **b** Validation of the c.262delG mutation in exon 2 by Sanger sequencing. The grey area indicates a frameshift mutation. **c** Validation for the c. G648T of exon 5 by Sanger sequencing. The grey area represents the mutation point

The exome sequencing results suggested that there was a synonymous mutation, c.648 G > T (p.L216 = , NM_000151.4), in exon 5 and a frameshift mutation, c.262delG (p.Val88Phefs*14, NM_000151.4), in exon 2 of the G6PC gene. According to the pedigree analysis results of first-generation sequencing, heterozygous mutations of c.648 G > T and c.262delG were derived from the patient’s father and mother (Fig. 2a-c). The location of these two mutations in the G6PC gene are a known mutation site of glycogen storage disease type Ia [18, 19].

**Fig. 3:**
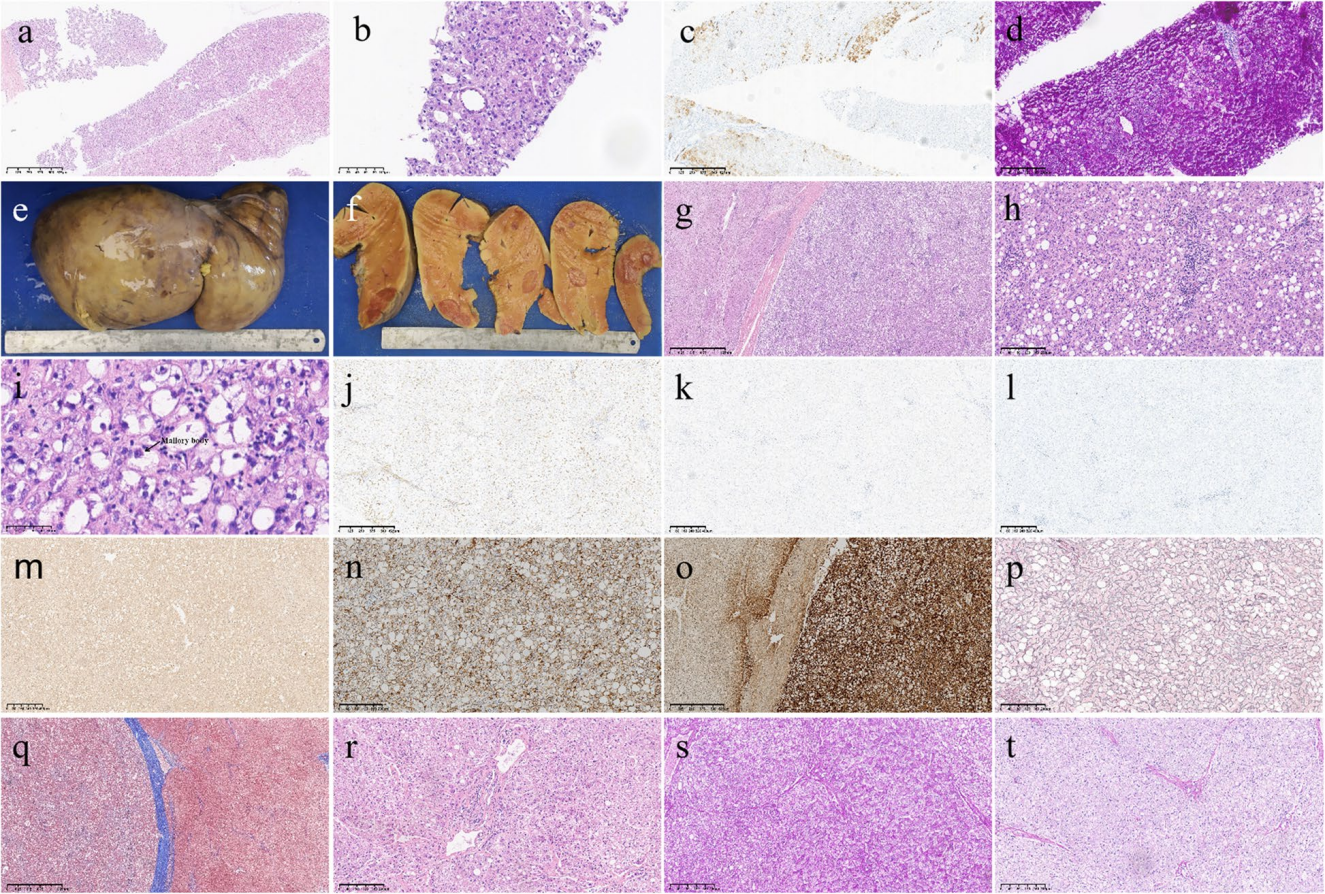
Pathological examination. **a** HE staining of liver biopsy tissue (20 ×). **b** At high magnification, an isolated arteriole is observed (400 ×). **c** CK7 showed slight proliferation of fine bile ducts (40 ×). **d** PAS staining of liver biopsy tissue suggested a large deposition of glycogen in hepatocytes (100 ×). **e–f** Gross image of the surgically resected liver. **g-i** HE staining of the adenoma tissue showed that the liver cells were arranged regularly against the background of fatty liver without atypia, the boundary between the normal liver tissue and the adenoma was clear, and the mallory body could be seen at high magnification (20 × -400 ×). **j** IHC showed positive CD34 staining of adenoma vascular endothelial cells, indicating incomplete vascularization (40 ×). **k** IHC showed negative GS staining in the adenomas (40 ×). **l** Ki-67 staining showing a proliferative rate of less than 2% in adenoma tissue (40 ×). **m** IHC showed no absence of L-FABP in the adenomas (40 ×). **n** IHC showed that beta-catenin was not activated in the adenomas (100 ×). **o** IHC shows that CRP is strongly and diffusely positive in adenomas (lower right) but not in normal liver tissue (upper left) (100 ×). **p** Foot’s staining methods showed that reticulin was not lost (100 ×).** q** Masson staining showing wide fibrous septa between normal liver tissue and adenoma tissue (40 ×). **r** HE staining of surrounding liver tissue (100 ×). **s** PAS staining of the specimen showed a large amount of glycogen deposition in the surrounding liver tissue (100 ×). **t** PAS-stained hepatocytes that were mostly digested by PAS-D (100 ×)

The liver biopsy results showed that several pieces of punctured liver tissue could be seen under the microscope, one of which was about the length of a liver lobule. Moreover, one isolated arteriole could be seen, and hepatocytes were arranged in a single row without atypia. CK7 showed slight proliferation of fine bile ducts and no evidence of malignancy. Adenomas or regenerated nodules were not excluded. In other liver tissues, the cytoplasm of hepatocytes was obviously loose and lightly stained, with ballooning degeneration and increased hepatocyte volume. PAS revealed steatosis of approximately 45% of hepatocytes, with several focal necroses in the hepatic lobules and incomplete hepatic plates. A few lymphocytes and monocytes infiltrated the portal area. The patient was diagnosed with mild chronic hepatitis combined with hepatocyte steatosis (Fig. 3a-d). Combined with the clinical and genetic test results, the pathological changes were consistent with those of GSD.

Finally, according to the clinical manifestations, auxiliary examinations, tissue pathology and genetic testing, this patient was considered to have GSD type Ia.

After surgical evaluation and patient family consent, the patient underwent living-donor liver allotransplantation (donor from her brother) under general anaesthesia. Postoperative gross pathology revealed that the volume and weight of the liver were significantly increased (V = 28.5 cm × 17 cm × 8 cm, W = 2050 g), showing a greyish-yellow and greyish-red colour, with protuberant nodules in some areas and several solid greyish-red nodules (φ = 0.2 cm-4.2 cm) in the section (Fig. 3e-f). Light microscopy revealed that the solid nodules were hepatocellular hyperplastic lesions, and there was no evidence of atypia or malignancy. Immunohistochemical results revealed positive expression of CD34 (incomplete vascularization), L-FABP, and CRP in solid nodule hepatocytes and negative expression of β-catenin, glypican 3 and GS. Ki-67 staining revealed a proliferative rate of less than 2% without reticulin loss. Masson staining revealed the formation of wide fibrous septa. TERT gene promoter mutations at locus 250 and locus 228 were not detected. These features suggest multiple inflammatory hepatic adenomas (Fig. 3g-q). The surrounding liver tissue showed watery degeneration of hepatocytes, cytoplasmic swelling of some hepatocytes, mixed steatosis of approximately 10% of hepatocytes, and fibrous tissue hyperplasia in the portal area with scattered chronic inflammatory cell infiltration (Fig. 3g). PAS staining was strongly positive. However, PAS-stained hepatocytes were mostly digested by periodic acid-Schiff-diastase (PAS-D) (Fig. 3r-t).

After surgery, the patient was transferred to the PICU for antirejection, anticoagulation, anti-infection and symptomatic support treatment. At discharge, the patient’s liver function recovered well. Imaging revealed no significant abnormalities in the bile duct or liver blood flow. Laboratory tests revealed that liver function and glucose levels were approximately normal (Table 1).

## Discussion and conclusions

GSD is a relatively rare inborn metabolic disorder caused by the accumulation of glycogen in different tissues. In 1929, von Gierke first described the clinical manifestations of GSD and the pathological changes in glycogen accumulation in the liver and kidney [20]. In 1952, Cori et al. reported that this disease was caused by a deficiency of glucose-6-phosphatase in the liver [21]. In 1995, Friedman et al. located the pathogenic gene G6PC at 17q21 [22]. G6PC is a key enzyme that maintains the stability of intermeal blood glucose. This enzyme catalyses the hydrolysis of G6P to glucose and phosphate in the last step of glycogen decomposition and gluconeogenesis; thus, the stability of blood glucose cannot be maintained in patients with GSD-Ia, which causes fasting hypoglycaemia. In turn, hypoglycaemia stimulates the bypass of G6P metabolism. This results in increased glycogen synthesis and excessive accumulation of glycogen in the liver and kidney and gradual liver and kidney enlargement. Moreover, a large amount of G6P accumulates in the cytoplasm, which leads to high cholesterol, triglyceride, uric acid and lactic acid levels [23]. In this case, the patient presented with baby face and growth retardation and multiple hypoglycaemia symptoms accompanied by obvious hepatomegaly. Laboratory examination revealed severe hyperlipidaemia and the deposition of lactic acid and uric acid, and imaging revealed multiple hepatic occupying lesions and fatty liver. These findings strongly suggest that the child was a GSD-Ia patient.

The G6PC gene is located on chromosome 17q21 and contains 5 exons. It encodes a 357 amino acid (36 kD) protein, and has a total of 9 transmembrane units. More than 100 mutations in the G6PC gene have been identified (http://www.HGMD.Cf.Ac.UK/ac/index.PHP) [24]. Genetic mutations at certain sites of G6PC are related to different ethnic groups. For example, the c.274C > T (p. Arg83Cys) and c.1039C > T (p. Q347X) mutations have a high incidence in Caucasian populations, whereas the c.648G > T mutation is most common in Japanese (91%) and Korean populations (75%) [23, 25, 26]. In China, the main mutations of G6PC are c.648G > T and c.248G > A (p. R83H) [27]. Interestingly, the c.648G > T mutation was mainly distributed in East China (80.95%), and the c.248G > A mutation was mainly concentrated in South China (22.5%) [27]. In this case, the patient was found to have two types of mutations, a synonymous mutation, c.648 G > T (p.L216 = , NM_000151.4), in exon 5 and a frameshift mutation, c.262delG (p.Val88Phefs*14, NM_000151.4), in exon 2 of the G6PC gene. The c.648 G > T mutation causes a change from CTG to CTT at protein 216. Although both codons encode leucine, this silent mutation creates a new splicing site 91 bp downstream of the authentic splice site. The c.262delG mutation is caused by the absence of guanine at nucleotide position 262, which causes a codon frame shift and changes the amino acid sequence of the protein. Moreover, a stop codon is generated at the 15th codon downstream of the protein, which truncates the protein by 254 amino acids and decreases enzyme activity.

GSD-Ia is usually associated with multiple hepatic adenomas (HCAs), but a few HCAs have been reported to be carcinogenic [28]. This requires pathologists to not only correctly identify the typical liver pathological changes of GSD but also determine the subtype of HCA and whether it is cancerous. The typical liver pathology of GSD is marked swelling of liver cells with fatty changes, and PAS staining suggests a large deposition of glycogen in hepatocytes. However, a large amount of glycogen accumulation in the liver can cause urea cycle disorders and glycogenic hepatopathy [29, 30]. Although the histological morphology under conventional light microscopy of these conditions is very similar to that of GSD, they have completely different clinical manifestations and pathogenesis. Urea cycle disorder is clinically visible as a toxic manifestation of hyperammonaemia. However, glycogenic hepatopathy usually occurs in the setting of poorly controlled diabetes and is responsive to diabetic control. Moreover, inflammatory adenomas (70%) and unclassified adenomas (30%) are the main types of glycogen storage disease adenomas [31]. The morphological findings of inflammatory adenomas include severe steatosis, sinusoidal dilatation and congestion, lymphocytic inflammation (generally mild and patchy), and pseudoportal portal tracts (or faux portal tracts) composed of an artery, bile duct-like proliferation, and a sleeve of connective tissue. IHC showed strong and diffuse positivity for CRP and/or SAA. Unclassified adenoma morphologies lack any unifying or distinguishing features, although some can exhibit fat and/or glycogen accumulation in tumour cells. These tumours cannot be classified into any other category of hepatic adenoma by immunohistological or molecular methods. Regardless of the type, the presence of HCC is indicated by the following changes: (1) there can be a distinct nodule within the adenoma that has a higher grade cytology than the background adenoma, with reticulin loss, increased proliferation, and other features of malignancy; (2) adenoma size > 5 cm; (3) β-catenin activation; (4) heavy lipofuscin pigment in adenoma; (5) androgen-associated adenomas; (6) myxoid morphology; and (7) TERT promoter mutation [31]. In this case, the morphologic features and IHC results of this patient were fully consistent with those of a GSD-Ia patient with multiple inflammatory hepatic adenomas, and no evidence of HCC was found.

The therapeutic goals for GSD-Ia patients are to maintain blood glucose within the normal range, correct metabolic disorders and reduce or delay the occurrence of serious complications. At present, nutritional therapy is still the most important method, and corn starch treatment is the most important method of nutritional therapy. The goal of treatment is to maintain blood glucose at ≥ 3.9 mmol/L [32]. Chronic complications can occur despite adherence to dietary therapy, and symptomatic treatment should be given to improve quality of life. For patients with hyperuricaemia and gout, sodium bicarbonate can be used to alkalize urine, and allopurinol or febuxostatin can inhibit uric acid synthesis. For patients with hyperlipidaemia, statins or beta blockers can be used to regulate lipids. Patients with granulocytopenia may be treated with granulocyte-stimulating factor. For patients with multiple hepatic adenomas, dynamic observation or liver transplantation can be performed [4, 33]. In this case, the patient was briefly treated with nutritional therapy after diagnosis and then underwent living-donor liver allotransplantation. After 14 months of follow-up, the patient recovered well, liver function and blood glucose levels remained normal, and no complications occurred.

However, our study did not detect the activity of related enzymes, especially the enzyme activity of liver tissue. Both electron microscopy and Gd-EOB-DTPA MRI are helpful in the diagnosis of GSDIa, but unfortunately, we have no relevant evidence [34, 35]. In addition, since the ovarian development and endocrine hormones of this patient have not been tested, we cannot sure whether GSDIa affects reproductive system development and endocrine homeostasis, which is worth further follow-up.

In this case, the patient was diagnosed with GSD-Ia combined with multiple inflammatory hepatic adenomas and received liver transplant treatment. For childhood patients who present with hepatomegaly, growth retardation, and laboratory test abnormalities, including hypoglycaemia, hyperuricaemia, and hyperlipidaemia, a diagnosis of GSD should be considered. Gene sequencing and liver pathology play important roles in the diagnosis and typing of GSD. When an adenoma is present, the pathologist accurately classifies it and assesses whether it is cancerous. Paediatricians and endocrinologists should immediately start treatment according to the patient’s condition, and liver transplant treatment is feasible if necessary.

## Data Availability

Additional data that support the findings of this study are available from the corresponding author.

## Abbreviations

GSD: Glycogen storage disease
WES: Whole-exome sequencing
IHC: Immunohistochemical
L-FABP: Liver fatty acid binding protein
CRP: C-reactive protein
GS: Glutamine synthetase;
G6PC: Glucose-6-phosphatase-α
HCC: Hepatocellular carcinoma
CT: Computerized tomography
